# The Evolution of Lesions on Follow-Up Magnetic Resonance Imaging of the Proximal Metacarpal Region in Non-Racing Sport Horses That Returned to Work (2015–2023)

**DOI:** 10.3390/ani14121731

**Published:** 2024-06-08

**Authors:** Elisabeth C. S. van Veggel, Katrien Vanderperren, Kurt T. Selberg, Hendrik-Jan Bergman, Brenda Hoogelander

**Affiliations:** 1 Sporthorse Medical Diagnostic Centre (SMDC), 5384 RC Heesch, The Netherlands; 2 Department of Morphology, Imaging, Orthopedics, Rehabilitation and Nutrition, Faculty of Veterinary Medicine, Ghent University, 9820 Merelbeke, Belgium; 3 Johnson Family Equine Hospital, College of Veterinary Medicine and Biomedical Sciences, Colorado State University, Fort Collins, CO 80523, USA; selberg@colostate.edu

**Keywords:** MRI, proximal suspensory ligament, proximal metacarpus, rescan, advanced imaging, equine

## Abstract

**Simple Summary:**

Proximal metacarpal pain is an important cause of lameness, and its diagnosis can be challenging. In some patients, magnetic resonance imaging (MRI) is needed for the evaluation of the proximal metacarpal region, and it is valuable for diagnosis. A challenge for these patients is to perform follow-up monitoring if the lesion cannot be visualized using radiography and/or ultrasonography, which is where rescan MRIs come into play. Various injuries have been visualized within the proximal metacarpal region, but it is currently unknown how they evolve in horses returning to soundness. This small case series evaluates various features of the proximal metacarpal region on MRI, which appear important to monitor, and includes hyperintense signals within the dorsal collagenous part of the proximal suspensory ligament (PSL) on T1W/T2*WGRE images, STIR hyperintense signals within the dorsal collagenous part of the PSL, and the third metacarpal bone (McIII). Complete normalization of the dorsal collagenous part of the PSL does not appear necessary for a return to soundness. A resolution of the McIII hyperintense STIR signal is expected for horses returning to soundness, and in this population of horses, there was a mean of 94 days (range: 47–202 days). A rescan time frame of 120 days for the proximal metacarpal region is suggested. The size of the proximal suspensory ligament does not change between the initial and follow-up MRI examinations.

**Abstract:**

Background: This study evaluates the change in an MRI of the proximal metacarpal region in a group of sport horses that returned to work. This retrospective analysis evaluated 18 limbs represented by 17 horses. Results: The hyperintense signal within the dorsal collagenous part of the proximal suspensory ligament (PSL) on T1W/T2*W GRE sequences decreased or stayed the same in the majority of cases. The hyperintense STIR signal within the dorsal collagenous part of the PSL resolved in the majority of the patients, and the third metacarpal bone (McIII) hyperintense STIR signal resolved in all patients. The dorsal margin irregularity of the PSL stayed the same, and McIII sclerosis and resorption of the palmar margin of McIII stayed the same in the majority of cases. McIII hyperintense STIR signal resolution carries a broad time range, with a mean of 94 days and a range of 47–202 days. Conclusions: Complete normalization of the dorsal collagenous part of the PSL does not appear necessary for a return to soundness, but a resolution of the McIII hyperintense STIR signal is expected for horses returning to soundness. A rescan period of 120 days for the proximal metacarpal region is suggested. In addition, there was no significant change in the size of the PSL between the initial and final MRI.

## 1. Introduction

Injury to the proximal metacarpal region is an important cause of forelimb lameness, with variable clinical signs and severity [1,2,3,4,5]. Radiography and ultrasonography are frequently used for evaluations of the proximal metacarpal region, as well as during follow-up examinations, as they are more readily available and less cost-inhibitive. There are limitations associated with these techniques, as the lesions identified may not show a good correlation with clinical lameness [6,7,8,9]. Previous reports suggest that magnetic resonance imaging (MRI) provides more detailed information than ultrasonography and that ultrasonographic findings should be interpreted with caution as ultrasonography is less precise and anatomically less detailed than MRI [6]. MRI has been used in the investigation of proximal metacarpal region pain and is an excellent diagnostic aid when other modalities fail to identify the cause of proximal metacarpal pain [10,11,12,13,14]. Concurrent bony and soft tissue injuries of the proximal metacarpal region are common, and the accurate diagnosis of all structures involved will help guide the selection of optimal therapy and estimate the prognosis [13,14]. Rescan MRIs have an impactful role in monitoring lesion progression, evaluating responses to treatment, and allowing for adjustments of the rehabilitation plan (workload and therapy) based on imaging and clinical findings [15]. The purpose of this study was to describe the follow-up MRI findings in sport (non-racing) horses who had their initial lameness located in the proximal metacarpal region and assess the evolution of the soft tissue and bone abnormalities of the proximal metacarpal region. In light of previous studies, the dorsal aspect of the proximal suspensory ligament as well as the STIR signal in McIII were expected to show the greatest lesions and change [13,14,16]. Lastly, it is hypothesized that the size of the PSL will stay constant between examinations.

## 2. Materials and Methods

Medical records from the Sporthorse Medical Diagnostic Centre (SMDC) were reviewed from September 2015 to September 2023. Cases were included in this retrospective analysis if they had at least 2 MRI examinations of the proximal metacarpal region and had their primary (initial) lameness located in the proximal metacarpal region, which resulted in a population of 18 limbs represented by 17 horses. This population of horses went back to full work after their last MRI and returned to a similar or higher level of performance. The lameness severity was available for a subset of horses and was variable, ranging from 1 to 3/5 on the AAEP gradation scale. These clinical examinations were not repeated prior to the MRI for those horses that were referrals. All MRI scans were performed within 1 week of their diagnostic examination. A total of 16 horses had diagnostic analgesia (direct infiltration of the PSL, blocked via the lateral palmar nerve or high four point nerve block). One horse received a scintigraphy (bone scan), which demonstrated regional increased radiopharmaceutical uptake in the palmaromedial aspect of the proximal metacarpus with subsequent diagnostic anesthesia (direction infiltration of the PSL), and one horse was scanned based on pain on palpation of the suspensory region. MRIs were performed using the standing Hallmarq system (0.27 Tesla). MRI grading analysis was performed on a minimum of transverse T1W GRE, T2*W GRE, T2W FSE, and STIR, FSE, as well as sagittal T1W GRE and dorsal T1W GRE of the proximal metacarpus, with additional scans used for interpretation if available. The images were retrospectively graded by ECVDI-LA diplomate (EVV) and ACVR and ACVR-EDI diplomate (KS) while being blinded to the horse identity and identification of the scan (initial or rescan). The grading scoring was used as previously described [14]. The pathology was graded from 0 (absent) to 3 (severe) for the following criteria: dorsal margin irregularity of the PSL, hyperintense signal within the dorsal collagenous part of the PSL on T1W/T2*W GRE within the suspensory ligament, hyperintense STIR signal within the dorsal collagenous part of the PSL, McIII hyperintense STIR signal, McIII low signal (sclerosis), and resorption of the palmar margin of the McIII. In addition, the presence or absence of a fissure/incomplete longitudinal fracture in McIII was noted. The last rescan MRI that was performed before the horse returned to full work/performance was used in the pattern of the grading scores as well as the days to return to work and comparison of the cross-sectional area (CSA) of the proximal suspensory ligament. This was selected as some horses had multiple rescans during their rehabilitation or resting period. Sub-specifications of the number of days for a resolution of the STIR hyperintense signal in McIII were noted. Return to work was classified as competing at the same or higher level at a similar frequency as prior to the injury for at least a minimum of 6 months. As was previously performed, the PSL size was measured at the most distal end of the SL attachment to the MCIII, where the dorsal margin of the SL became distinguishable from the palmar cortex of the McIII, approximately 2 and 3 cm distal to the carpometacarpal joint [6,14,17]. The CSA encompassed the entirety of the PSL (both medial and lateral lobes) in the transverse section. 

The analyzed variables will be described as means and medians with their respective minimum and maximum ranges. A statistical analysis was performed on the CSA size of the PSL, and paired *t*-tests (two-tailed *t*-test) were applied. The CSA of the PSL at the first and last MRIs were used in the comparison. It was hypothesized that the size of the PSL would be unchanged (a null hypothesis) between the two timepoints. Statistical analysis was performed using Graphpad Prism version 10.1.1 for Mac GraphPad Software, Boston, MA, USA, www.graphpad.com (accessed on 1 May 2024).

Ethical review and approval were not required for the animal study as this was a retrospective study with clinical patients. The owner of the horse or person responsible for the horse signed a consent agreement for the use of anonymous data for research when registering the patient at the clinic. The standing low-field MRI imaging parameters used during examinations of the proximal metacarpal region in this study are listed in Table 1. 

## 3. Results

**Figure 1:**
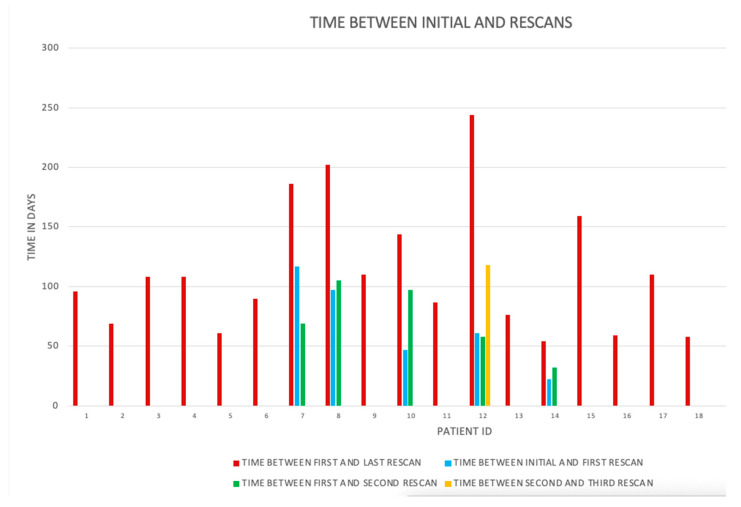
Graphic representation of the time between the initial and rescan MRIs for the 18 limbs.

**Figure 2:**
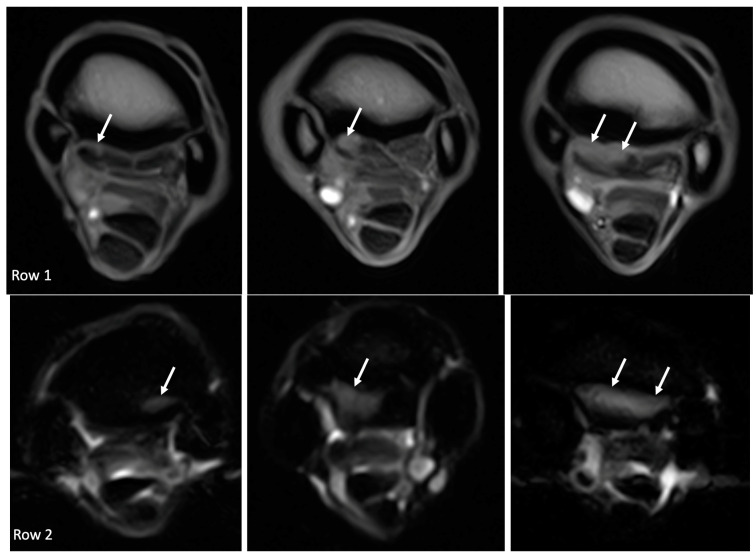

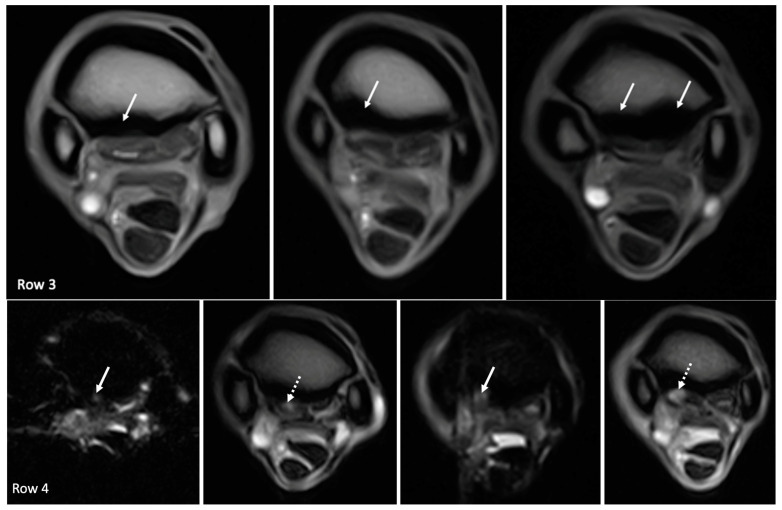
Composite figure with examples of the gradations: in row 1, hyperintense signal within the dorsal collagenous part of the proximal suspensory ligament, as seen on T1W GRE transverse images (from left to right, grade 1, grade 2, and grade 3, as indicated by the white arrows); in row 2, McIII STIR hyperintense signal on STIR FSE transverse images (from left to right, grade 1, grade 2, and grade 3, as indicated by the white arrows); in row 3, McIII sclerosis on T1W GRE transverse images (from left to right, grade 1, grade 2, and grade 3, as indicated by the white arrows); and in row 4, PSL STIR hyperintense signal in the dorsal collagenous part of the proximal suspensory ligament on STIR FSE transverse images (grade 1 and 2 as indicated by the white arrows), and the corresponding T2*W GRE transverse images with dotted arrows (also grade 1 and 2).

The image quality was good in all scans, and no MRIs were excluded based on image quality. Eighteen limbs represented by seventeen horses met the inclusion criteria for return to work, with one horse having bilateral lameness. The group was represented by 8 right front (RF) limbs and 10 left front (LF) limbs. There were six geldings, six mares, and five stallions. The one horse with bilateral front limb lameness was a gelding used for driving. The remainder were composed of 5 dressage horses and 11 showjumpers. In the dressage horses, there were three grand prix dressage horses, one FEI-level pony, and one lower-level dressage horse. In the showjumpers, there were three grand prix-level showjumpers, seven at 1.40 m or a higher level, and one at a 1.30 meter level. The age ranged from 4 to 13 years, with a mean age of 9.2 years and a median age of 9 years. The range of days between the initial scan and the last MRI scan was 54–244 days, with a mean of 112 days (Figure 1). The horse with the bilateral front limb was re-scanned within the same time frame for both proximal metacarpi at 108 days. Within this group that returned to soundness, there were five patients who had multiple rescans (four horses had three scans and one horse had four scans) (Figure 1). The number of limbs and percentage of the findings within each group and the change in the (pathological) findings per limb are displayed in Table 2, and an example for the gradation for hyperintense signal within the dorsal collagenous part of the PSL on T1W/T2*W GRE within the suspensory ligament, hyperintense STIR signal within the dorsal collagenous part of the PSL, McIII hyperintense STIR signal, and McIII low signal (sclerosis) is given in Figure 2.

**Figure 3:**
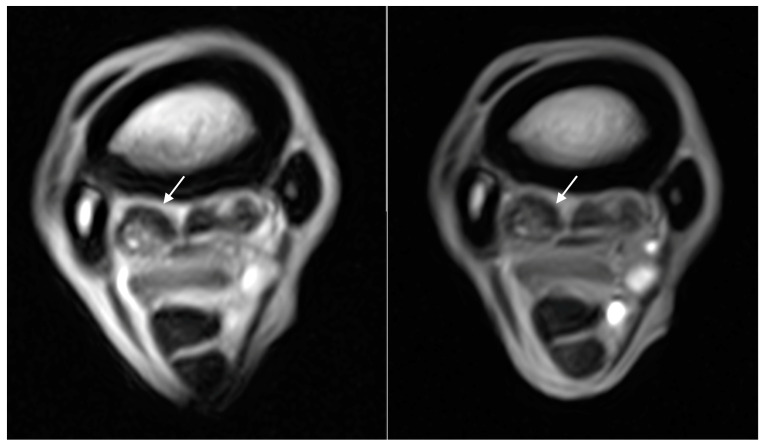
Transverse T1W GRE images (right side is medial) of the initial (**left image**) and rescan (**right image**) MRI with a similar grade 1 hyperintense signal, as highlighted by the white arrow, within the dorsal collagenous part of the medial lobe of the RF proximal suspensory ligament in a 6-year-old dressage pony stallion. The time between scans was 61 days, and the pony returned to full work.

**Figure 4:**
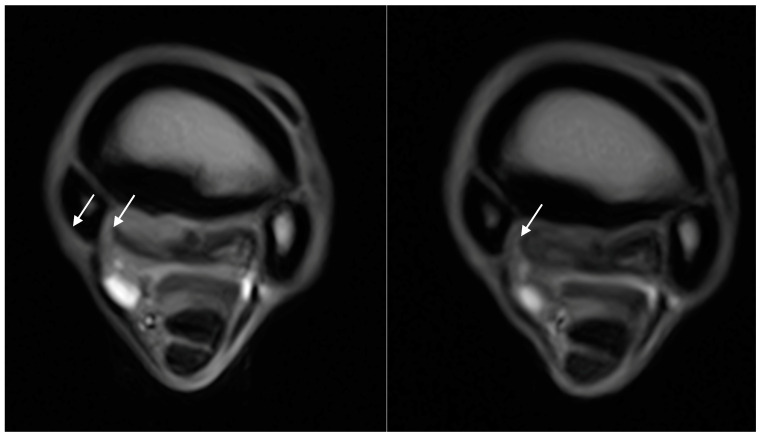
Transverse T1W GRE images (left side is medial) of the initial (**left image**) and rescan (**right image**) MRI in a 7-year-old showjumping mare. Initially, there was a grade 3 hyperintense signal within the dorsal collagenous part of the medial lobe of the LF proximal suspensory ligament, which decreased to a grade 1 in the rescan, as highlighted by the white arrow. The time between scans was 202 days, and the horse returned to full work.

**Figure 5:**
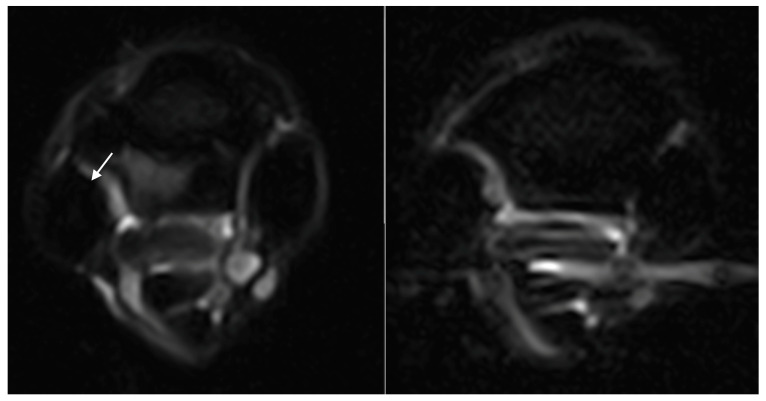
Transverse STIR FSE images (right side is medial) of the initial (**left image**) and rescan (**right image**) MRI in 6-year-old dressage pony stallion. There is a decrease in STIR hyperintense signal within the RF proximal McIII from a grade 2 (**left image**) to a grade 0 (**right image**). The STIR hyperintense signal is indicated by the white arrow in the left image. The time between scans was 61 days, and the pony returned to full work.

**Figure 6:**
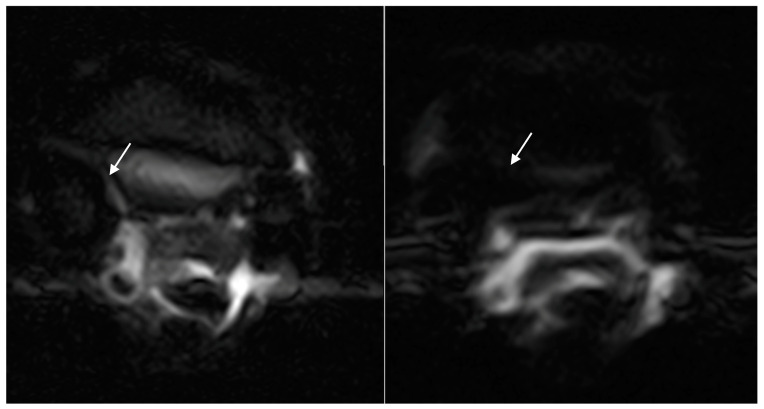
Transverse STIR FSE images (right side is lateral) of the initial (**left image**) and first rescan (**right image**) MRI in a 6-year-old showjumping stallion. Note the decrease in STIR hyperintense signal within the LF proximal McIII from a grade 3 to a grade 1 after 22 days, as indicated by the white arrows in both images. The STIR hyperintense signal resolved completely after a further 32 days (54 days from the initial scan), and the horse returned to full work.

The number of limbs with each gradation of the lesions in the initial and final MRI scans is displayed in Table 3. Dorsal margin irregularity of the PSL was present in 12 limbs and stayed similar in all cases. The hyperintense signal within the dorsal collagenous part of the medial lobe PSL on T1W/T2*W GRE was present in ten limbs, and it remained similar in four limbs, decreased by one grade in four limbs, decreased by two grades in one limb, and increased by one grade in one limb (Figure 3 and Figure 4). The hyperintense signal within the dorsal collagenous part of the medial and lateral lobe PSL on T1W/T2*W GRE was present in one limb, and it decreased by one grade. In none of the horses did the hyperintense signal within the dorsal collagenous part of the PSL on T1W/T2*W GRE images completely resolve. The hyperintense STIR signal within the dorsal collagenous part of the medial lobe of the PSL was present in five limbs. It resolved in four limbs (decreased two grades in three limbs and one limb by one grade) and remained identical in one limb. The McIII hyperintense STIR signal was present in 16 limbs and resolved in all limbs by the last rescan (Figure 5 and Figure 6). The bilateral limb patient is included in this count of 16 limbs. The distribution of the McIII hyperintense STIR signal was eight medial, five medial, and lateral and three lateral. At the initial MRI, there were five limbs with grade one, three limbs with grade two, and eight limbs with grade three. When looking at the earliest time point (applicable to those patients with multiple rescans), where the McIII hyperintense STIR signal was resolved, there was a mean of 94 days and a range of 47–202 days. All limbs had some level of McIII low signal (sclerosis), and this remained constant, except for one horse with bilateral lameness, which had a bilateral fissure/incomplete longitudinal fracture of the palmaromedial cortical bone of the proximal metacarpus on the primary MRI. No fissure/incomplete longitudinal fracture of the proximal metacarpus was present in the other horses. The distribution of the sclerosis was medial in 15 limbs, medial and lateral in 2 limbs, and central to medial in 1 limb. Resorption of the palmar margin of the McIII was present in 12 limbs, stayed the same in 11 limbs, and increased by one grade in 1 limb. For the horses with multiple rescans (five horses), there was gradual improvement in at least one gradation of the pathology (soft tissue and/or bone) as time progressed, which was encouraging for further rescans. None of the horses with multiple rescans had an increased gradation of a lesion (soft tissue or bone) at any time point. Furthermore, no limbs developed new lesions (soft tissue or bone) at the time of the rescans, and the lesions stayed localized to the same region (medial, lateral, medial and lateral, or central).

The CSA of the PSL was not statistically different between the initial and last MRI (*p* value = 0.3755, *p* > 0.05). The mean of the initial CSA of PSL was 2.86 cm^2^ (min = 2.14 cm^2^, max = 3.62 cm^2^, and median = 2.91 cm^2^). The mean of the rescan (last) CSA of PSL was 2.80 cm^2^ (min = 2.22 cm^2^, max = 3.6 cm^2^, and median = 2.73 cm^2^).

## 4. Discussion

This study aims to evaluate the change in MRI of the proximal metacarpus and the proximal suspensory ligament in a group of sport horses that returned to work/performance. In general, rescan MRI examinations are important to follow-up lesions that are identified on MRIs, especially those that are not able to be visualized on radiography and ultrasonography [6,7,8,9]. As previously published, the hyperintense STIR signal within the proximal McIII or dorsal collagenous part of the PSL and the hyperintense signal within the dorsal collagenous part of the PSL on T1W GRE and T2*W GRE images identified on MRI are considered clinically relevant findings [14]. The hyperintense STIR signal within McIII was resolved in all patients. This hyperintense STIR signal noted in McIII can represent transient edema/inflammation, osteonecrosis, osteofibrosis, bone marrow edema, hyperemia, and/or hemorrhage [18,19]. It has been proposed that hyperintense STIR signals associated with trauma (acute, chronic, or repetitive) typically resolve with rest, while degeneration results in persistently high signal intensity and may result in chronic lameness [17,18,19]. Similarly to a previous case study on racing thoroughbreds, a resolution of the hyperintense STIR signal was observed in those patients that returned to work successfully [20]. Furthermore, similar to our result, a resolution of the hyperintense STIR signal is expected in those horses returning to soundness. The reported time for the resolution of hyperintense STIR signals in humans is highly variable, ranging from 3 weeks to 2 years [21,22,23]. When looking at the earliest time point where the McIII hyperintense STIR signal was resolved, there was a mean of 94 days (range of 47–202 days) in our population of horses. On the basis of our small number of cases, we suggest that if a hyperintense STIR signal in McIII is not resolved by approximately 6 months and there is no improvement in the gradation and/or amount of the hyperintense STIR signal, the prognosis may be negatively affected. This is similar to previous publications, where the majority of follow-up scans occurred in the first 6 months [24,25,26,27]. Thus, the suggested timeframe from this case series is in line with previous studies that suggest that the mean resting period required for proximal suspensory ligament/metacarpal injury would be 3–6 months [28]. The severity of the hyperintense STIR signal did not appear to influence the ability to return to work, as even the grade 3 hyperintense STIR signal resolved successfully. This is in agreement with the previously reported data that indicated that the severity of the lesions does not appear to influence the prognosis [14].

The hyperintense signal within the dorsal collagenous part of the PSL on T1W and T2*W GRE images stayed the same or decreased in the majority of patients. No patients had complete resolution of their hyperintense signal on T1W and T2*W GRE images; therefore, complete resolution does not appear necessary for a return to full work in this small population of horses. The hyperintense signal on T1W and T2*W GRE images may represent degeneration, tearing, and/or fibrosis [14].

The hyperintense STIR signal within the dorsal collagenous part of the PSL resolved in all but one limb, where it remained static. Interestingly, in a previous study, there was an association between resolution of the hyperintense STIR signal within tendons and ligaments and return to soundness, but not in bones [29,30]. However, both of these studies covered injuries to the distal limb, and it was noted that the number of horses with bone marrow lesions was low and the sample size was too small for statistical evaluation [30]. 

Although not part of this group of horses who were assessed in this study, there were an additional four horses that did not return to work successfully but that received rescan MRIs of the proximal metacarpal region. The interesting features in these rescan MRIs were that 75% of these horses did not have a resolution of the STIR hyperintense signal in McIII and that the hyperintense signal on T1W and T2*W GRE within the dorsal collagenous part of the PSL worsened in all cases. Future work on the progression of lesions on MRIs in those horses returning to work vs. those remaining lame is necessary to confirm the trend seen. 

The dorsal margin irregularity of the PSL stayed the same. As previously mentioned, this is often seen in normal horses, and considering their static findings, this may be a normal patient finding or a low-field MRI artifact [12,31]. 

The McIII sclerosis stayed the same, except for the horse with the bilateral fissure/incomplete longitudinal fracture of the palmaromedial cortex of the proximal metacarpus. Increased sclerosis after the healing of the fissure/incomplete longitudinal fracture was expected. A recent study found that the palmar cortex of the McIII and McIII sclerosis was unchanged after training [32].

The palmar cortical resorption of the McIII was unchanged in the majority of the horses. A recent study indicated that in hindlimbs, the proximal third metatarsal bone shows individual morphological variations, with longitudinal linear ridges [33]. Possibly similar findings could be seen in the front limbs, inducing a concave/undulating palmar cortical margin of the McIII that mimics resorption. Alternatively, palmar cortical resorption of the McIII may be an MRI finding that takes time to occur and, therefore, is less likely to change within the timeframe of the rescans. Lastly, the hypothesis that the size of the PSL will stay constant between MRI examinations was correct, as there was no statistical difference between the size of the initial and (last) rescan measurements. Furthermore, the CSA of the PSL measured falls in line with the previously published normal ranges [6,14]. This is similar to a recent publication that observed that there was no significant difference between pre- and post-season measurements and that six months of endurance training were not sufficient to induce changes in the thickness of the suspensory ligament on low-field MRI images [32]. In addition, a study in hind limbs found that the CSA of the PSL was not influenced by plantar fasciotomy and neurectomy of the deep branch of the lateral plantar nerve [34].

Overall, the range of re-scanning was 54–244 days, with a mean of 112 days. When selecting the time for rescanning, the clinical examination often guided the timing, and patients that had not yet improved in their clinical examination were less likely to be scheduled for rescan MRIs than those patients already showing an improvement. Based on the small numbers available, a suggested rescan time of the proximal metacarpal region is 120 days, guided by the McIII STIR signal, as this covers 13/15 patients’ resolution of the McIII STIR signal. A 90-day rescan period covered 8/15 patients’ resolution of the McIII STIR signal. If there is a reduction in the STIR McIII but not yet complete resolution, another rescan MRI is recommended at 60-day intervals.

The limitations of our study included the small number of patients as well as the non-standardized group of patients, which may have strongly impacted our conclusions, so further clinical follow-up studies with higher patient numbers would be valuable. Another limitation is the large number of veterinarians involved in the caseload, which resulted in non-conform therapeutic treatments as well as non-conform subjective lameness assessments. Therefore, the therapeutic treatments were not compared in relation to the MRI findings. Furthermore, the previously used grading system in a previously published study by the same authors was a limitation in this study as, unfortunately, it did not always allow for noted improvements, which would be visible subjectively [14]. An example would be the subjective decrease in the hyperintense signal on T1W GRE/T2*W GRE images, which would appear to be an improvement, but based on the grading system, this follow-up scan would receive a similar grade when the size/extent of distribution stayed the same. Furthermore, the rating system was based on the assessment of the subjective grading of two radiologists. 

Future studies, combining both MRI and CT imaging for assessment of the proximal metacarpal region would be useful and complementary, as each imaging modality has its strengths. 

## 5. Conclusions

Suggested areas to monitor in rescan MRIs of the proximal metacarpal region appear to be the hyperintense signal within the dorsal collagenous part of the PSL on T1W/T2*W GRE images, the STIR hyperintense signal within the dorsal collagenous part of the PSL, and the McIII STIR hyperintense signal, as these parameters change the most between the initial and follow-up MRIs. In this group of horses, complete normalization of the dorsal collagenous part of the PSL did not appear necessary for a return to soundness, but a resolution of the McIII hyperintense STIR signal is expected in horses returning to soundness. The McIII hyperintense STIR signal resolution had a mean of 94 days and a range of 47–202 days. Based on the small numbers available, a suggested rescan time for the proximal metacarpal region is 120 days. There was no significant change in the size of the PSL between the initial and final MRI in this group of horses. 

## Figures and Tables

**Table 1:** Standing low-field MRI parameters used during examination of the proximal metacarpal region in this study.

Sequence	TR (ms)	TE (ms)	Flip Angle (Deg.)	FOV (cm)	Matrix Size	Slice Thickness (mm)	Gap (mm)	Fr × Ph
T1W GRE	52	8	50	170 × 170	256 × 256	5	0.6	170 × 170
T2*W GRE	68	13	28	170 × 170	256 × 256	5	0.6	170 × 160
T2W FSE	1500	110	90	170 × 170	256 × 256	5	0.6	128 × 144
STIR FSE	1834	22	90	170 × 170	256 × 256	5	0.6	128 × 144

TR: repetition time; TE: echo time; FOV: field of view; Fr × Ph: number of frequency encoding and phase encoding steps.

**Table 2:** The number of limbs and percentage of the findings and the change in the (pathological) findings per limb.

Finding	Number of Limbs and Percentage with Findings	Resolved	Decreased	Unchanged	Increased
Dorsal margin irregularity of the PSL	12/18 (75%)	-	-	12/12 (100%)	-
Hyperintense signal on T1W GRE and T2*W GRE within the dorsal collagenous part of the PSL	11/18 (61%)	0/11	6/11 (55%)	4/11 (36%)	1/11 (9%)
Hyperintense STIR signal within the dorsal collagenous part of the PSL	5/18 (28%)	4/5 (80%)	-	1/5 (20%)	-
McIII hyperintense STIR signal	16/18 (89%)	16/16 (100%)	-	-	-
McIII sclerosis	18/18 (100%)	-	-	16/18 (89%)	2/18 (11%)
McIII palmar cortical resorption	12/18 (67%)	-	-	11/12 (92%)	1/12 (8%)

**Table 3:** The number of limbs with each gradation (0–3) for the various findings assessed of the proximal metacarpal region in the initial and rescan MRI. In those patients where multiple scans were performed, the last MRI was noted.

Finding	Initial MRI	(Last) Rescan MRI
Dorsal margin irregularity of the PSL	Grade 0: 5 limbs Grade 1: 7 limbs Grade 2: 6 limbs Grade 3: 0 limbs	Grade 0: 5 limbs Grade 1: 7 limbs Grade 2: 6 limbs Grade 3: 0 limbs
Hyperintense signal on T1W GRE and T2*W GRE within the dorsal collagenous part of the PSL	Grade 0: 7 limbs Grade 1: 5 limbs Grade 2: 5 limbs Grade 3: 1 limb	Grade 0: 7 limbs Grade 1: 10 limbs Grade 2: 1 limbs Grade 3: 0 limbs
Hyperintense STIR signal within the dorsal collagenous part of the PSL	Grade 0: 13 limbs Grade 1: 1 limb Grade 2: 4 limbs Grade 3: 0 limb	Grade 0: 17 limbs Grade 1: 0 limbs Grade 2: 1 limbs Grade 3: 0 limbs
McIII hyperintense STIR signal	Grade 0: 2 limbs Grade 1: 5 limbs Grade 2: 3 limbs Grade 3: 8 limbs	Grade 0: 18 limbs Grade 1: 0 limbs Grade 2: 0 limbs Grade 3: 0 limbs
McIII sclerosis	Grade 0: 0 limbs Grade 1: 11 limbs Grade 2: 6 limbs Grade 3: 1 limb	Grade 0: 0 limbs Grade 1: 10 limbs Grade 2: 6 limbs Grade 3: 2 limbs
McIII palmar cortical resorption	Grade 0: 7 limbs Grade 1: 8 limbs Grade 2: 3 limbs Grade 3: 0 limbs	Grade 0: 6 limbs Grade 1: 9 limbs Grade 2: 3 limbs

## Data Availability

Further inquiries can be directed to the corresponding author.

## References

[1] Denoix J., Perrot B., Bousseau B., Sciciuna C. (1991). Images Échographiques Des Lesions Du Muscle Interosseoux III (Ligament Suspenseur Du Boulet). Prat. Vet. Equine.

[2] Gibson K.T., Steel C.M. (2002). Conditions of the Suspensory Ligament Causing Lameness in Horses. Equine Vet. Educ..

[3] Meehan L., Labens R. (2016). Diagnosing Desmitis of the Origin of the Suspensory Ligament. Equine Vet. Educ..

[4] Dyson S.J. (2003). Proximal Metacarpal and Metatarsal Pain: A Diagnostic Challenge. Equine Vet. Educ..

[5] Dyson S. (2007). Diagnosis and Management of Common Suspensory Lesions in the Forelimbs and Hindlimbs of Sport Horses. Clin. Tech. Equine Pract..

[6] Bischofberger A.S., Konar M., Ohlerth S., Geyer H., Lang J., Ueltschi G., Lischer C.J. (2006). Magnetic Resonance Imaging, Ultrasonography and Histology of the Suspensory Ligament Origin: A Comparative Study of Normal Anatomy of Warmblood Horses. Equine Vet. J..

[7] Werpy N.M., Denoix J.-M. (2012). Imaging of the Equine Proximal Suspensory Ligament. Vet. Clin. N. Am. Equine Pract..

[8] Werpy N.M., Denoix J.M., McIlwraith C.W., Frisbie D.D. (2013). Comparison between Standard Ultrasonography, Angle Contrast Ultrasonography and Magnetic Resonance Imaging Characteristics of the Normal Equine Proximal Suspensory Ligament. Vet. Radiol. Ultrasound.

[9] Denoix J.-M., Coudry V., Jacquet S. (2008). Ultrasonographic Procedure for a Complete Examination of the Proximal Third Interosseous Muscle (Proximal Suspensory Ligament) in the Equine Forelimbs. Equine Vet. Educ..

[10] Brokken M.T., Schneider R.K., Sampson S.N., Tucker R.L., Gavin P.R., Ho C.P. (2007). Magnetic Resonance Imaging Features of the Proximal Metacarpal and Metatarsal Injuries in the Horse. Vet. Radiol. Ultrasound.

[11] Powell S.E., Ramzan P.H.L., Head M.J., Shepherd M.C., Baldwin G.I., Steven W.N. (2010). Standing Magnetic Resonance Imaging Detection of Bone Marrow Oedema-type Signal Pattern Associated with Subcarpal Pain in 8 Racehorses: A Prospective Study. Equine Vet. J..

[12] Nagy A., Dyson S. (2012). Magnetic Resonance Imaging Findings in the Carpus and Proximal Metacarpal Region of 50 Lame Horses. Equine Vet. J..

[13] Murray R.C., Tranquille C.A., Walker V.A., Milmine R.C., Bak L., Tacey J.B., Bolas N.M. (2020). Magnetic Resonance Imaging Findings in the Proximal Metacarpal Region of 359 Horses and Proximal Metatarsal Region of 64 Horses Acquired under Standing Sedation. J. Equine Vet. Sci..

[14] van Veggel E., Selberg K., van der Velde-Hoogelander B., Bolas N., Vanderperren K., Bergman H.J. (2021). Magnetic Resonance Imaging Findings of the Proximal Metacarpal Region in Warmblood Horses: 36 Lame and 26 Control Limbs (2015–2021). Front. Vet. Sci..

[15] Werpy N.M. (2012). Recheck Magnetic Resonance Imaging Examinations for Evaluation of Musculoskeletal Injury. Vet. Clin. N. Am. Equine Pract..

[16] Barrett M.F., Manchon P.T., Hersman J., Kawcak C.E. (2018). Magnetic Resonance Imaging Findings of the Proximal Metacarpus in Quarter Horses Used for Cutting: Retrospective Analysis of 32 Horses 2009–2012. Equine Vet. J..

[17] Nagy A., Dyson S. (2009). Magnetic Resonance Anatomy of the Proximal Metacarpal Region of the Horse Described from Images Acquired from Low- and High-Field Magnets. Vet. Radiol. Ultrasound.

[18] Murray R.C., Blunden T.S., Schramme M.C., Dyson S.J. (2006). How Does Magnetic Resonance Imaging Represent Histologic Findings in the Equine Digit ?. Vet. Radiol. Ultrasound.

[19] Dyson S., Blunden T., Murray R. (2012). Comparison between Magnetic Resonance Imaging and Histological Findings in the Navicular Bone of Horses with Foot Pain. Equine Vet. J..

[20] Mizobe F., Nomura M., Kato T., Nambo Y., Yamada K. (2017). Signal Changes in Standing Magnetic Resonance Imaging of Osseous Injury at the Origin of the Suspensory Ligament in Four Thoroughbred Racehorses under Tiludronic Acid Treatment. J. Equine Sci..

[21] Costa-Paz M., Muscolo D.L., Ayerza M., Makino A., Aponte-Tinao L. (2001). Magnetic Resonance Imaging Follow-up Study of Bone Bruises Associated with Anterior Cruciate Ligament Ruptures. Arthrosc. J. Arthrosc. Relat. Surg..

[22] Roemer F., Bohndorf K. (2002). Long-Term Osseous Sequelae after Acute Trauma of the Knee Joint Evaluated by MRI. Skelet. Radiol..

[23] Mandalia V., Fogg A.J.B., Chari R., Murray J., Beale A., Henson J.H.L. (2005). Bone Bruising of the Knee. Clin. Radiol..

[24] Gold S.J., Werpy N.M., Gutierrez-Nibeyro S.D. (2017). Injuries of the Sagittal Groove of the Proximal Phalanx in Warmblood Horses Detected with Low-Field Magnetic Imaging: 19 Cases (2007–2016). Vet. Radiol. Ultrasound.

[25] Faulkner J.E., Joostens Z., Broeckx B.J.G., Hauspie S., Mariën T., Vanderperren K. (2023). Follow-Up Magnetic Resonance Imaging of Sagittal Groove Disease of the Equine Proximal Phalanx Using a Classification System in 29 Non-Racing Sports Horses. Animals.

[26] Dyson S., Nagy A., Murray R. (2011). Clinical and Diagnostic Imaging Findings in Horses with Subchondral Bone Trauma of the Sagittal Groove of the Proximal Phalanx. Vet. Radiol. Ultrasound.

[27] Ramzan P.H.L., Powell S.E. (2010). Clinical and Imaging Features of Suspected Prodromal Fracture of the Proximal Phalanx in Three Thoroughbred Racehorses. Equine Vet. J..

[28] Bertone A.L., Baxter G.M. (2011). Suspensory Ligament Desmitis. Adams and Stashak’s Lameness in Horses.

[29] Vanel M., Olive J., Gold S., Mitchell R.D., Walker L. (2012). Clinical Significance and Prognosis of Deep Digital Flexor Tendinopathy Assessed over Time Using MRI. Vet. Radiol. Ultrasound.

[30] Holowinski M., Judy C., Saveraid T., Maranda L. (2010). Resolution of Lesions on STIR Images Is Associated with Improved Lameness Status in Horses. Vet. Radiol. Ultrasound.

[31] Werpy N.M. (2007). Magnetic Resonance Imaging of the Equine Patient: A Comparison of High- and Low-Field Systems. Clin. Tech. Equine Pract..

[32] Likon I., Dyson S., Nagy A. (2023). Magnetic Resonance Imaging Measurements of the Proximal Palmar Cortex of the Third Metacarpal Bone and the Suspensory Ligament in Non-Lame Endurance Horses before and after Six Months of Training. Animals.

[33] Dancot M., Joostens Z., Audigié F., Busoni V. (2023). The Plantar Proximal Cortex of the Third Metatarsal Bone Shows Raised Longitudinal Ridges at the Suspensory Ligament Enthesis in Normal Equine Isolated Limbs—A Radiographic, Computed Tomography, and MRI Study. Front. Vet. Sci..

[34] Scharf A., de Solis C.N., Sampson S.N., Glass K., Watts A.E. (2022). Suspensory Ligament Size Does Not Change after Plantar Fasciotomy and Neurectomy of the Deep Branch of the Lateral Plantar Nerve by Ultrasonographic Assessment. Vet. Surg..

